# The Safety and Immunogenicity of a 13-Valent Pneumococcal Polysaccharide Conjugate Vaccine (CRM197/TT) in Infants: A Double-Blind, Randomized, Phase III Trial

**DOI:** 10.3390/vaccines12121417

**Published:** 2024-12-16

**Authors:** Zhiqiang Xie, Jiangjiao Li, Xue Wang, Lili Huang, Jinbo Gou, Wei Zhang, Haitao Huang, Wangyang You, Feiyu Wang, Xiaolong Li, Jinming Zhang, Qiang Han, Xiaomin Ma, Yanxia Wang

**Affiliations:** 1 Henan Province Center for Disease Control and Prevention, Zhengzhou 450003, China; xiezqshang@163.com (Z.X.); 13643826177@163.com (L.H.); zwzzu@163.com (W.Z.); dsrt12345@163.com (W.Y.); 2 National Institutes for Food and Drug Control, Beijing 102629, China; lijiangjiao@nifdc.org.cn; 3 CanSino Biologics Inc., Tianjin 300457, China; xue.wang@cansinotech.com (X.W.); jinbo.gou@cansinotech.com (J.G.); haitao.huang@cansinotech.com (H.H.); feiyu.wang@cansinotech.com (F.W.); xiaolong.li@cansinotech.com (X.L.); jinming.zhang@cansinotech.com (J.Z.); qiang.han@cansinotech.com (Q.H.)

**Keywords:** 13-valent pneumococcal polysaccharide conjugate vaccine, phase III trial, opsonophagocytic killing assay, infants

## Abstract

Objectives: This study aimed to evaluate the immunogenicity and safety of a 13-valent pneumococcal polysaccharide conjugate vaccine (CRM197/TT) (PCV13i) in infants. Methods: A total of 1200 infants were randomly assigned to either the experimental PCV13i group or the control PCV13 group in a 1:1 ratio. Each group received a three-dose series of the vaccine at 2, 4, and 6 months of age, followed by a booster dose at 12–15 months. Blood samples were collected before and 30 days after both primary and booster vaccinations. The primary immunogenicity endpoints were the seropositive rate and the geometric mean concentration (GMC) of IgG antibodies against the 13 pneumococcal serotypes. The primary safety endpoint was the incidence of adverse reactions within 0–7 days and 0–30 days after vaccination. Results: Results showed that the experimental PCV13i was well tolerated, with a safety profile comparable to that of the control vaccine. Following primary vaccination, the GMCs of IgG responses against serotypes 1, 5, 6A, 6B, 14, and 18C in the experimental group were lower than those in the control group, while responses against serotypes 3, 4, 7F, 9V, 19A, 19F, and 23F were higher. The experimental group exhibited higher opsonophagocytic killing assay (OPA) geometric mean titers (GMTs) for serotypes 3, 7F, 19A, and 19F compared to the control group, while GMTs for serotypes 1, 5, 6A, and 18C were lower. Following booster vaccination, OPA GMTs of the experimental group remained higher than those of the control group for serotypes 3, 7F, and 19F, while GMTs for serotype 5 were lower. Both vaccines induced robust immune responses, with high seropositive rates and significant increases in antibody levels following vaccination. Conclusions: The experimental PCV13i demonstrated non-inferiority to the control PCV13 in terms of immunogenicity.

## 1. Introduction

Pneumococcal disease remains a significant global health burden, particularly among infants and young children, despite advances in medical care and vaccine development. Streptococcus pneumoniae, a leading cause of morbidity and mortality worldwide, is associated with a range of clinical manifestations, from mild upper respiratory tract infections to severe invasive diseases such as pneumonia, meningitis, and bacteremia [1,2].

Pneumococcal conjugate vaccines (PCVs) have significantly reduced the incidence of pneumococcal disease and its associated complications [3]. PCV13 is composed of polysaccharides from 13 different pneumococcal serotypes conjugated to a carrier protein, promoting a T cell-dependent immune response and long-lasting immunogenicity [4]. PCV13 works by eliciting a protective immune response against the included pneumococcal serotypes. When administered, the immune system recognizes the polysaccharide antigens conjugated to the carrier protein, leading to the production of antigen-specific antibodies and memory B cells [5]. This immune response provides both humoral and cellular immunity against invasive pneumococcal infections, thereby reducing the risk of disease transmission and associated morbidity and mortality.

Recent clinical trials have provided valuable insights into the efficacy, safety, and mechanism of action of PCV13 in various populations and settings. For instance, a clinical study compared the immunogenicity and safety between PCV13 and PCV7 in infants, showing PCV13 was as effective as PCV7 in preventing pneumococcal disease caused by the seven common serotypes and offered expanded protection against the six additional serotypes. The PCV13 safety profile was comparable to that of PCV7 [6]. Another study evaluated the efficacy and safety of PCV13 in a large cohort of elderly adults, demonstrating significant reductions in vaccine-type pneumococcal pneumonia and invasive pneumococcal disease compared to placebo [7]. A study evaluated the administration of PCV13 to older infants and children without prior pneumococcal vaccination. The results indicated catch-up schedules of PCV13 could induce anti-pneumococcal immune responses for protection [8].

In China’s National Immunization Program, a PCV as an important childhood vaccine that is readily available in the private market has not been included yet [9]. However, with the high demand for safe and effective PCVs, there are three PCV13 products that have been approved by the national medical products administration, produced by Pfizer Inc. (New York, NY, USA), Minhai Biotechnology Co., Ltd. (Beijing, China), and Walvax Biotechnology Co., Ltd. (Kunming, China), respectively. Previously, a phase I study was performed in infants aged above 2 months (minimum 6 weeks old) to observe the preliminary safety of a 13-valent pneumococcal polysaccharide conjugate vaccine (CRM197/TT) (PCV13i) produced by CanSino Biologics Inc. (Tianjin, China) (NCT04100772). In this manuscript, we present the methodology and results of our phase III clinical trial, focusing on the immunogenicity and safety outcomes of PCV13i in a cohort of infants. By elucidating the vaccine’s performance in this population and integrating findings from recent clinical trials, we aim to contribute to the existing body of evidence supporting the use of PCV13i.

## 2. Methods

### 2.1. Study Plan and Participants

This was a randomized, double-blind phase III trial. It was conducted in three study sites of Henan province, China, between April 2021 and September 2022. Eligible participants aged 2 months (minimum 6 weeks) without previous pneumococcal vaccination history were recruited. Exclusion criteria for the first dose included the following: preterm birth (delivery before the 37th week of pregnancy); low birth weight (<2300 g for girls, <2500 g for boys); a history of dystocia, asphyxia rescue, and nervous system damage; axillary body temperature before vaccination >37.0 °C; congenital malformations or developmental disorders, genetic defects, severe malnutrition; a history of epilepsy, convulsions, or a family history of psychosis; having received immune-boosting or inhibitor therapy within the past 3 months (continuous oral or infusion for more than 14 days); having been diagnosed with congenital or acquired immunodeficiency, HIV infection, lymphoma, leukemia or other autoimmune diseases; asplenia caused by any condition, defects in spleen function; known or suspected diseases including severe respiratory disease, severe cardiovascular disease, liver and kidney diseases, skin diseases, malignant tumors; in the acute phase of a chronic disease; history of coagulation abnormalities (e.g., coagulation factor deficiency, coagulation disorders); a history of severe allergic reaction to vaccination; and other conditions judged by the investigator to be unsuitable for participation in this clinical trial. Those who had a severe allergic reaction after a previous vaccination dose or serious adverse reactions with a causal relationship with the previous vaccination dose were excluded from follow-up vaccinations.

A total of 1200 participants were randomly assigned to either an experimental group, which received the PCV13i experimental vaccine, or a control group, which received the PCV13 control vaccine, in a 1:1 ratio. Both experimental and control vaccines were administered in three doses at 2, 4, and 6 months for primary immunization, followed by a booster dose at 12–15 months. The protocol and informed consent were approved by the ethics committee of Henan Provincial Center for Disease Control and Prevention (2018-YM-006–02). Written informed consent was obtained before screening.

### 2.2. Vaccines and Mask

The vaccines were administered intramuscularly in a volume of 0.5 mL (lot number: PCV202009002C). The experimental vaccine was a 13-valent pneumococcal polysaccharide conjugate vaccine (CRM197/TT) produced by CanSino Biologics Inc. It consisted of polysaccharides from 13 serotypes of *Streptococcus pneumoniae* (1, 3, 4, 5, 6A, 6B, 7F, 9V, 14, 18C, 19A, 19F, and 23F), which were obtained through fermentation and purification. These polysaccharides were conjugated with CRM197 (1, 3, 4, 5, 6A, 6B, 9V, 14, 18C, 19A, and 23F) or TT (7F and 19F) carrier proteins, and the conjugated solution of each type was mixed and adsorbed onto aluminum phosphate adjuvant. The control PCV13 was manufactured by Pfizer Pharmaceuticals (lot number: DK0537DK2065). 

Statistical randomizers were randomized using Stata 15.1/SE software. The study numbers for 2-month-old (minimum 6-week-old) participants were A0001~A1200, and they were randomly assigned to the experimental group or the control group according to the ratio of 1:1, with 600 subjects in each group. Randomization and blinding were achieved through vaccine blinding. The sponsor provided the experimental and control vaccines, which had been tested and approved, and commissioned the statistical unit to conduct the blinding. The randomizer of the statistical unit used Stata 16./SE software to randomly blind the vaccines using the method of block randomization, and the vaccine numbering rule was ‘Y + 4-digit running number’. The vaccines were randomly assigned to the experimental group and control group by consecutive numbering. The results of the randomized grouping of the subjects and the random allocation of the vaccines were correlated with the 2 letter codes X and Y. The random allocation of the vaccines to the experimental and control groups was performed using the ‘Y + 4 digit running number’ rule. The statistician imported the results of subject randomization and vaccine randomization blinding into the central randomization system, which was used for vaccine access control and vaccine allocation during the study. After successful screening of subjects, researchers at each site participating in this clinical trial logged in to the central randomization system to obtain the study number of the subject and the corresponding vaccine number at the same time. For example, if a subject with site 01 number A0001 was randomized to site 01 with letter code X, the central randomization system allocated the vaccine from the vaccines allocated to site 01. When the vaccine was assigned, the central randomization system randomly selected the vaccine with the letter code X from the vaccines assigned to Site 01 for vaccination.

In order to maintain blindness throughout the trial, the correspondence between participant numbers and vaccine numbers was known only to the unblinded researchers, and was sealed after the assignment was completed. Unblind researchers were required not to participate in the clinical trial or disclose the contents of the blinding to any person participating in the clinical trial.

### 2.3. Outcomes

Primary immunogenicity endpoints were seropositive rate (antibody concentration ≥0.35 µg/mL) and geometric mean concentration (GMC) of IgG antibody 30 days after 3 doses of primary immunization and 30 days after booster immunization, while the primary safety endpoint was incidence of adverse reactions within 0~7 days and 0~30 days after each vaccination dose. Secondary immunogenicity endpoints included the geometric mean titer (GMT) and the ratio of titer ≥ 1:8 for specific serotype in pneumococcal opsonophagocytic killing assay (OPA) antibody 30 days after 3 doses of primary immunization and 30 days after booster immunization in some selected participants, and the ratio of serotype-specific pneumococcal IgG antibody concentration ≥1.0 µg/mL 30 days after 3 doses of primary immunization and 30 days after booster immunization. Secondary safety endpoint was the incidence of serious adverse events (SAEs) from the first dose to 6 months after the third dose during the primary immunization and 6 months after the booster immunization (safety observation period).

### 2.4. Statistical Analysis

Sample size was calculated based on the assumption of non-inferiority of antibody positive rate. According to the results of the preclinical trial of the control vaccine, the antibody positive rate of the control group was set at 87% after 3 doses of immunization, with α = 0.025 (one-sided) and a total power (1-β) = 0.90. Given 13 serotypes, the probability of class II error was adjusted to prevent inflation, resulting in β = 0.10/13 = 0.00769, and the non-inferiority margin = −0.1, with p0 = 0.87. A z test was chosen for calculating the test statistic. The experimental and control groups were based on a 1:1 design. Using these parameters, the Non-Inferiority of Two Independent Proportions test in NCSS-PASS software (22.0) determined a sample size requirement of 455 individuals per group Anticipating a dropout rate of 20%, this necessitated 569 cases per group. To accommodate site assignment and blinding, 600 participants were enrolled in both the experimental and control groups.

Safety data between groups were compared using the $\chi^{2}\;$ test, corrected $\chi^{2}$ test, or Fisher’s exact probability method. For immunogenicity analysis, antibody titers were log-transformed, and *t*-tests were utilized when assumptions of normality and variance homogeneity were met, while corrected *t*-tests were applied when normality was present but variance was not aligned, specifically to assess GMCs at each time point. The proportion of serotype-specific pneumococcal IgG antibodies ≥0.35 µg/mL and ≥1.0 µg/mL for each serotype at each time point was calculated separately, and comparisons between the groups were made by using the $\chi^{2}$ test, corrected $\chi^{2}$ test, or Fisher’s exact probability method. *t*-tests and corrected *t*-tests as mentioned above were utilized to compare GMTs of each serotype-specific OPA, and between-group comparisons of proportions of OPA titers ≥1:8 were performed using either the $\chi^{2}$ test, corrected $\chi^{2}$ test, or Fisher’s exact probability method.

## 3. Results 

**Figure 1:**
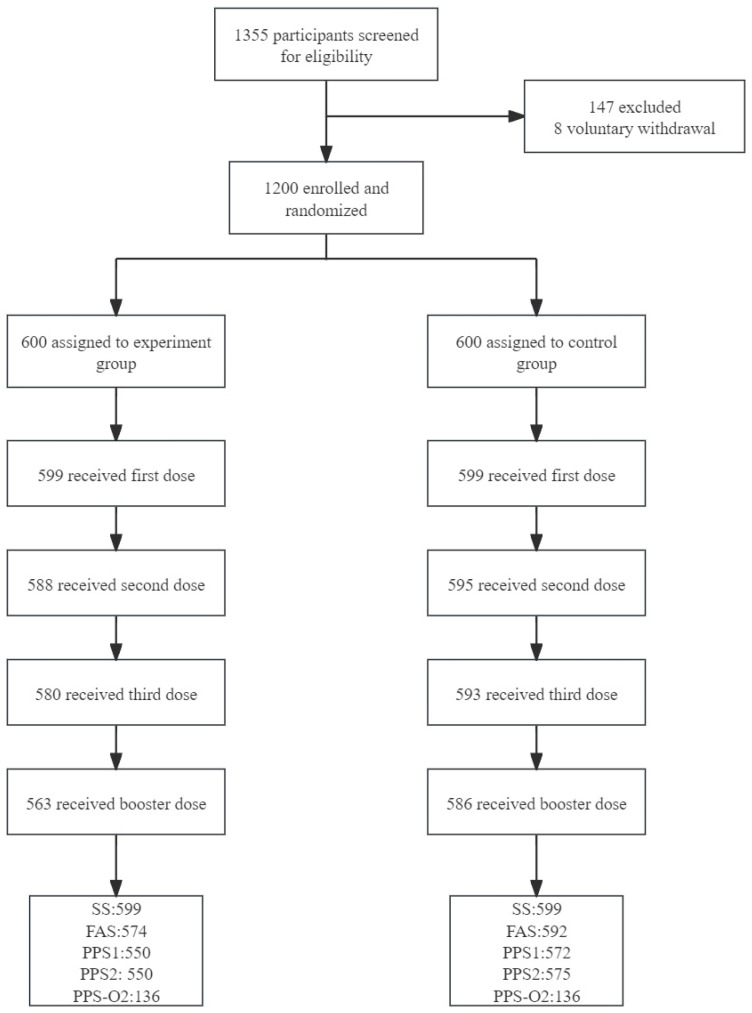
Trial profile. Note: SS represents safety set. FAS represents full analysis set. PPS1 is a subset of per protocol set, which contains 2-month-old subjects who completed 3 vaccination doses and had pre- and post-3-dose IgG test results. PPS2 is also a subset of the per protocol set, which contains 2-month-old subjects completing the 4th vaccination with pre- and post-immunization IgG test results. PPS-O2 included the participants in PPS2 with post immunization opsonophagocytic killing assay results.

In total, 1200 infants were randomly assigned to an experimental and control group with the ratio of 1:1. In each group, 599 participants received the first dose of either experimental PCV13i or control PCV13. Among those, 580 participants completed the three-dose primary immunization with the experimental vaccine, and blood samples were collected from 573 participants 30 days after primary immunization. In the control group, 593 participants completed the three-dose primary immunization, and blood samples were collected from 592 participants 30 days after primary immunization. Totals of 563 participants in the experimental group and 586 participants in the control group completed booster vaccination (Figure 1). Except for a significantly higher percentage of males in the experimental group than in the control group (53.42% vs. 47.08%, *p* = 0.028), baseline demographics including age, body mass index (BMI) and axillary temperature before the first dose were comparable (Table 1).

The overall adverse events in the experimental group and control group were 517 (29.26) and 466 (26.08), respectively, with a significant difference between the groups (*p* = 0.034). The overall adverse reactions in the experimental and control group after primary vaccination were 389 (22.01%) and 365 (20.43%), while they were 118 (20.96%) and 129 (22.01%) after booster vaccination. Common symptoms in both groups included redness as the predominant local adverse reaction and fever as the predominant systemic adverse reaction. After three vaccination doses, there was a significant difference in swelling between the experimental and control groups (55 [3.11] vs. 34 [1.90], *p* = 0.021). After booster vaccination, the incidence of redness in the experimental group was higher than that in the control group (34 [6.04%] vs. 18 [3.07%]), and the difference was statistically significant (*p* = 0.016). In both groups, the adverse reactions within 30 days after primary vaccination and booster vaccination were predominantly systemic symptoms and essentially grade 1 or 2. Grade 3 adverse reactions were exclusively systemic, occurring at a low incidence of less than 1.00%. No SAEs reported during the safety observation period were related to the vaccine in both groups. There were no statistically significant differences in the incidence of total adverse reactions, solicited adverse reactions, and unsolicited adverse reactions within 30 days after vaccination between the experimental and control groups (Table 2). Overall, the participants tolerated the adverse reactions, which typically resolved within 1–2 days without treatment.

Table 3 illustrates the outcomes between the experimental and control groups for 13 serotypes before and after primary vaccination in terms of seropositive rate, GMC, and GMI. For serotype 1 of IgG, the seropositive rates were 100% in both groups following primary vaccination. The GMC was lower in the experimental group compared to the control group after primary vaccination (2.63 vs. 3.18, *p* < 0.001). For serotypes 3 and 4, the experimental group exhibited higher GMC and GMI compared to the control group after primary vaccination. Serotype 5’s IgG responses pre-primary vaccination outcomes indicated significant differences between the groups, with the experimental group having lower seropositive rates and GMCs, but post-primary, both groups achieved high seropositive rates, with substantial increases in GMC and GMI. The control group showed higher GMCs after primary vaccination compared to the experimental group for serotypes 6A and 6B. Serotypes 14 and 23F showed high post-primary seropositive rates and substantial increases in GMC and GMI in both groups, with no significant differences for GMI between the experimental and control groups for these serotypes. Serotypes 7F, 9V, 19A, and 19F exhibited notable differences, with the experimental group achieving significantly higher GMIs, indicating substantial increases in antibody levels compared to the control. However, the control group showed significantly higher GMC (2.53 vs. 1.61, *p* < 0.001) and GMI (10.74 vs. 8.15, *p* = 0.008) for serotype 18C compared to the experimental group.

Table 4 shows the seropositive rates and GMCs of IgG responses in the experimental and control groups before and after booster vaccination across 13 serotypes. For all serotypes, both groups showed substantial increases in GMC after booster vaccination, with the seropositive rates reaching 100% except for serotype 3, 5 and 18C. For serotype 3, 19A and 19F, there were significant differences in GMC either before or after booster vaccination (*p <* 0.001). The GMIs of these serotypes in the experimental group were 22.71, 17.44 and 22.90, respectively, significantly higher than those in the control group, which were 14.78, 11.27 and 9.23 (all *p <* 0.001). Serotype 4 also demonstrated effective vaccination responses. The GMC of the experimental group improved from 0.59 to 3.35, resulting in a GMI of 65.49. The control group, starting with the GMC of 0.55, reached 2.57 after booster vaccination, with a GMI of 40.66. For serotype 7F, the experimental group achieved a GMI of 49.63, which was significantly higher than that of the control group (21.70, *p <* 0.001). The control group began with a lower baseline GMC of 1.38, which increased to 4.94, resulting in a GMI of 21.70. This indicated that although both groups showed notable improvements, the experimental group exhibited significantly higher increases in GMC. The experimental group’s GMC increased from 0.87 to 3.70, resulting in a GMI of 16.81 for serotype 9V. In comparison, the control group saw GMC rise from 0.69 to 3.31, with a GMI of 13.13. The experimental group demonstrated a significantly higher post-booster GMI (*p* = 0.021).

Table 5 shows data comparing the immune response of OPA between the experimental and control groups after primary and booster vaccination, measured by GMT and the percentage of participants with OPA ≥ 1:8. After primary vaccination, the GMTs against serotypes 1, 3, 7F, 19A, and 19F were significantly higher in the experimental group compared to the control group. The experimental group showed significantly lower GMTs against serotypes 5, 6A, and 18C compared to the control after primary vaccination, particularly for serotype 5 (100.57 vs. 480.13, *p* < 0.001). The experimental group showed higher GMTs against serotypes 4, 9V, 14, and 23F, whereas the control group showed a higher GMT against 6B, with no significant difference between the experimental and control groups for these serotypes. Additionally, for most serotypes, more than 94% of participants in both groups achieved the desired immune response, with no significant differences observed between groups except for serotype 5, where the experimental group had a lower percentage (93.38% vs. 100.00%, *p* = 0.007). After booster vaccination, except for serotype 7F in the control group and serotype 14 in the experimental group, most serotypes showed a great increase in GMT in both the experimental and control groups, suggesting an enhanced response to booster vaccination. Serotypes 3, 7F, and 19F in the experimental group showed significantly higher GMTs than those in the control group (all *p <* 0.001). However, the GMT against serotype 5 was significantly lower in the experimental group (241.31 vs. 572.43, *p* < 0.001), while the same trend was observed in the percentage of participants with OPA ≥ 1:8 (94.12% vs. 100.00%, *p* = 0.012).

## 4. Discussion

The results of this phase III trial indicated the PCV13i is safe and immunogenic in 2-month-old (minimum 6-week-old) infants. The safety profile of the experimental PCV13i was comparable to that of the control PCV13, with the majority of adverse events being mild and self-limiting. The most common local adverse reaction was redness, and the most common adverse reaction was fever, all of which were generally mild and resolved without intervention. After primary vaccination, the incidence of swelling in the experimental group (3.11%) was significantly higher than that in the control group (1.90%, *p* = 0.021), and after booster vaccination, the incidence of redness in the experimental group (6.04%) was significantly higher than that in the control group (3.07%, *p* = 0.016). Notably, the incidence of SAEs was low, and none were deemed related to the vaccination. This aligns with the existing literature on the safety of PCV13, further supporting its suitability for widespread use [10,11]. 

One notable finding was the varying immune response to specific serotypes. Following primary vaccination, the experimental group showed higher immune responses to serotypes 3, 7F, 19A, and 19F compared to the control group combined with the results of the ELIAS and OPA assay. With regard to the distribution of pneumococcal serotypes, the WHO organized the first national investigation of the serotype distribution of S. pneumoniae in the hinterland of China, which encompassed 27 hospitals and institutes in 18 provinces and cities in 1981. The study revealed that the eight common serotypes were 5, 6, 1, 19, 2, 14, 23, and 3, accounting for 63.3% of all isolates. The most prevalent serotypes causing lung infection in infants were 1 and 14, while those isolated from older children and adults with lung infections were 1 and 5. Due to the severe overuse of antibiotics in China, the resistance of S. pneumoniae to various antibiotics is on the rise. A series of studies from several locations in China after 2000 indicated that the distribution of common serotypes changed to 19A, 19F, 23F, 6B, 15B, and 14. [12]. From 2010 to 2015, Zhang et al.’s study [13] identified 3, 14, 19A, 19F, and 23F as common serotypes that caused invasive pneumococcal disease (IPD) in both Chinese children and adults. Then, a systematic review concluded that 6B, 14, 19F, 19A, and 23F were the most prevalent serotypes among under-fives in China in 2020 [14]. The PCV13i covering serotypes 1, 3, 4, 5, 6A, 6B, 7F, 9V, 14, 18C, 19A, 19F, and 23F showed comparable immunogenicity with the control group. This suggests that the experimental PCV13i may offer enhanced protection against these serotypes, which not only contribute significantly to global morbidity and mortality, but also are more consistent with the epidemiological trend of pneumonia [5]. In addition, the higher immune responses to serotypes 7F and 19F showed the advantages of the TT vector. Pre-existing immunity to TT subsequently could enhance the immunogenicity of TT-conjugated pneumococcal vaccine, as verified in a mouse model. Some infant studies also revealed that the immunogenicity of PCV11-DT and PCV7/13-CRM197 was enhanced in participants previously vaccinated with DTP vaccine [15]. Similarly, the advantages of TT and CRM vectors may be more significant in the case of co-administration. Additionally, one study assessed the clinical efficacy and effectiveness of 19F-containing vaccines against 19A disease or nasopharyngeal carriage and found that immunization with 19F-containing pneumococcal conjugate vaccines can provide some direct protection against 19A disease [16]. Therefore, the cross-protection between serotypes may enhance the immunogenicity of PCV13i.

Enhanced responses to these serotypes could lead to better overall protection and potentially reduce the incidence of IPDs in vaccinated populations. However, for serotype 5, the control group demonstrated higher GMC and GMI values, indicating a potentially weaker response from the experimental vaccine to this specific serotype. This discrepancy may warrant further investigation into the formulation or administration protocols of the experimental vaccine to ensure optimal effect across all included serotypes. Further, the high seropositive rates after booster vaccination and substantial increases in the GMC of IgG antibodies observed in both the experimental and control groups indicate that the experimental PCV13i is effective in inducing a robust immune response. This finding is consistent with previous studies, which have demonstrated the capability of PCV13 to generate strong immunogenicity across various pneumococcal serotypes [17,18]. The robust immune responses elicited by the experimental PCV13i, characterized by high seropositive rates and significant increases in GMC, underscore its potential efficacy and effectiveness in protecting against pneumococcal diseases. Both the experimental and control vaccines achieved nearly 100% seropositivity for most serotypes after booster vaccinations, and the experimental PCV13i demonstrated non-inferiority to the control PCV13. Such high immunogenicity may be crucial for the protection of infants and young children, who are particularly vulnerable to severe pneumococcal infections such as pneumonia, meningitis, and bacteremia [19]. 

Except for serotype 5, all 12 other serotypes elicited an immune response in 100% of participants with OPA ≥ 1:8 after booster vaccination, demonstrating that a high proportion of participants attained protective antibody levels with both experimental and control vaccines. In addition, most serotypes showed an increase in GMT in the experimental group post-booster, indicating that the booster dose is effective in enhancing the immune response. The booster dose, administered at 12–15 months, resulted in insignificant increases in antibody titers for both the experimental and control groups. The booster dose is essential for maintaining high levels of immunity and may extend protection into later childhood. 

The widespread use of PCV13 not only protects vaccinated individuals but also contributes to herd immunity, indirectly protecting unvaccinated individuals by reducing the transmission of vaccine-covered pneumococcal serotypes [3]. This is particularly important in low-resource settings with incomplete vaccine coverage [20]. Herd immunity effects have been well-documented in countries with established PCV immunization programs, leading to declines in IPD among both vaccinated and unvaccinated populations [4]. One of the strengths of this study is its randomized, double-blind design, which minimizes bias and enhances the reliability of the results. The large sample size and multicenter approach contribute to the robustness of the findings. However, there are some limitations to note. The study focused exclusively on infants aged 2 months, while immunity effects of PCV13i in other age groups were also evaluated in the clinical trial, with results anticipated in a forthcoming paper. Furthermore, future research may focus on the long-term immunogenicity and efficacy of the experimental PCV13i by monitoring vaccinated cohorts over several years to gather valuable data on the immune durability and the necessity of additional booster doses. Moreover, with the evolving landscape of pneumonia disease and changing population needs, the types and quality of pneumonia vaccines available on the market are continually advancing, exemplified by emerging polyvalent pneumonia vaccines like PCV15, PCV20 and PCV24 designed to address a broader range of pneumonia serotypes [21,22,23,24], highlighting the urgent need for more accessible polyvalent pneumonia vaccines covering prevalent serotypes.

## 5. Conclusions

This phase III trial provides compelling evidence that the PCV13i is both safe and highly immunogenic in infants. The study demonstrated that the experimental PCV13i induced strong immune responses against multiple pneumococcal serotypes, achieving high seropositive rates and substantial increases in antibody concentrations after vaccination. Importantly, the safety profile of the experimental PCV13i was comparable to that of the control vaccine, with most adverse events being mild and self-limiting, and no vaccine-related SAEs were reported. The robust immunogenicity and favorable safety profile of the experimental PCV13i make it a promising candidate for widespread use, potentially enhancing protection against a broader range of pneumococcal serotypes and contributing to the reduction of pneumococcal disease burden globally. Continued surveillance and long-term studies are essential to fully understand the durability of the immune response and the long-term impact of PCV13i. Further research should also focus on evaluating the effect of PCV13i in diverse populations. By addressing these aspects, we can ensure that PCV13i continues to provide robust protection and contributes to ongoing efforts to combat pneumococcal disease. 

## Figures and Tables

**Table 1:** Baseline demographics of study population (SS).

Characteristics	Experimental Group (*n* = 599)	Control Group (*n* = 599)	*p*
Age (months)	2.20 ± 0.43	2.19 ± 0.44	0.735
Gender			**0.028**
Male (%)	53.42	47.08	
Female (%)	46.58	52.92	
BMI (kg/m^2^)	16.92 ± 1.97	16.79 ± 2.10	0.253
Axillary temperature before first dose (°C)	36.72 ± 0.31	36.70 ± 0.31	0.263

Note: Significant differences (*p* < 0.05) are shown in bold. BMI represents body mass index.

**Table 2:** Overall profiles of adverse reactions 0–30 days post-primary and booster vaccination.

	Post-Primary Vaccination			Booster Vaccination		
Reaction *n* (%)	Experimental Group (*n* = 1767)	Control Group (*n* = 1787)	*p*	Experimental Group (*n* = 563)	Control Group (*n* = 586)	*p*
Overall adverse events	517 (29.26)	466 (26.08)	**0.034**	154 (27.35)	167 (28.50)	0.665
Overall adverse reactions	389 (22.01)	365 (20.43)	0.247	118 (20.96)	129 (22.01)	0.664
Grade 3	2 (0.11)	4 (0.22)	0.693	2 (0.36)	4 (0.68)	0.719
Unsolicited adverse reactions	2 (0.11)	4 (0.22)	0.693	3 (0.53)	4 (0.68)	>0.999
Solicited adverse reactions	388 (21.94)	364 (20.37)	0.246	116 (20.60)	127 (21.67)	0.658
Redness	86 (4.87)	69 (3.86)	0.142	34 (6.04)	18 (3.07)	**0.016**
Pain	8 (0.45)	4 (0.22)	0.240	0	1 (0.17)	>0.999
Induration	5 (0.28)	0	0.071	0	0	-
Swelling	55 (3.11)	34 (1.90)	**0.021**	8 (1.42)	10 (1.71)	0.697
Decreased appetite	5 (0.28)	1 (0.06)	0.215	0	0	-
Cough	24 (1.36)	23 (1.29)	0.853	9 (1.60)	14 (2.39)	0.339
Grade 3	1 (0.06)	1 (0.06)	>0.999	0	3 (0.51)	0.262
Fever	227 (12.85)	240 (13.43)	0.607	74 (13.14)	94 (16.04)	0.165
Grade 3	0	2 (0.11)	0.500	2 (0.36)	1 (0.17)	0.972
Irritable	28 (1.58)	27 (1.51)	0.859	1 (0.18)	2 (0.34)	>0.999
Nausea	1 (0.06)	1 (0.06)	>0.999	0	0	-
Vomiting	14 (0.79)	10 (0.56)	0.397	2 (0.36)	0	0.240
Diarrhea	29 (1.64)	24 (1.34)	0.463	6 (1.07)	4 (0.68)	0.703
Grade 3	1 (0.06)	0	0.497	0	0	-

Note: Significant differences (*p* < 0.05) are shown in bold. The “*n*” in post-primary vaccination is the sum of participants who completed three doses of primary vaccination.

**Table 3:** Type-specific seropositive rates, GMC and GMI of IgG response pre- and post-primary vaccination (PPS2).

		Pre-Primary Vaccination				Post-Primary Vaccination					
Serotype	Group	Seropositive Rate % (95%CI)	*p*	GMC (95% CI)	*p*	Seropositive Rate % (95% CI)	*p*	GMC (95% CI)	*p*	GMI (95% CI)	*p*
1	Experiment	43.90 (39.70–48.16)	0.393	0.19 (0.17–0.22)	0.216	100.00 (99.33–100.00)	-	2.63 (2.48–2.78)	**<0.001**	13.53 (11.82–15.50)	0.401
	Control	46.43 (42.30–50.61)		0.22 (0.19–0.24)		100.00 (99.36–100.00)		3.18 (3.01–3.35)		14.68 (12.85–16.76)	
3	Experiment	4.55 (2.97–6.65)	0.519	0.06 (0.05–0.07)	0.558	98.54 (97.15–99.37)	**<0.001**	1.16 (1.10–1.22)	**<0.001**	19.79 (17.10–22.91)	**<0.001**
	Control	5.39 (3.69–7.57)		0.06 (0.05–0.07)		89.74 (86.96–92.10)		0.75 (0.72–0.79)		12.11 (10.43–14.06)	
4	Experiment	8.56 (6.36–11.22)	0.071	0.05 (0.04–0.06)	0.052	99.82 (98.99–100.00)	0.488	2.56 (2.43–2.71)	**<0.001**	49.84 (42.24–58.80)	**<0.001**
	Control	11.83 (9.30–14.75)		0.06 (0.05–0.07)		100.00 (99.36–100.00)		2.13 (2.02–2.25)		33.61 (28.64–39.45)	
5	Experiment	26.41 (22.77–30.31)	**0.004**	0.15 (0.13–0.17)	**0.032**	94.90 (92.71–96.58)	**<0.001**	1.00 (0.94–1.05)	**<0.001**	6.58 (5.78–7.49)	**0.009**
	Control	34.26 (30.38–38.30)		0.18 (0.16–0.20)		98.78 (97.51–99.51)		1.52 (1.44–1.60)		8.36 (7.37–9.47)	
6A	Experiment	33.88 (29.93–38.01)	**0.025**	0.21 (0.19–0.24)	0.113	99.82 (98.99–100.00)	>0.999	2.37 (2.23–2.52)	**<0.001**	11.14 (9.76–12.72)	0.072
	Control	40.35 (36.31–44.49)		0.24 (0.22–0.27)		99.83 (99.03–100.00)		3.20 (3.00–3.41)		13.26 (11.57–15.20)	
6B	Experiment	65.03 (60.87–69.02)	0.135	0.42 (0.38–0.46)	**0.048**	100.00 (99.33–100.00)	>0.999	3.15 (2.98–3.34)	**<0.001**	7.57 (6.80–8.43)	0.568
	Control	69.22 (60.87–69.02)		0.47 (0.43–0.52)		99.83 (99.03–100.00)		3.76 (3.52–4.01)		7.93 (7.05–8.92)	
7F	Experiment	43.72 (39.52–47.98)	0.391	0.21 (0.18–0.24)	0.295	100.00 (99.33–100.00)	-	10.51 (9.89–11.18)	**<0.001**	50.83 (43.98–58.74)	**<0.001**
	Control	46.26 (42.13–50.43)		0.23 (0.20–0.26)		100.00 (99.36–100.00)		5.61 (5.31–5.92)		24.64 (21.45–28.31)	
9V	Experiment	52.46 (48.19–56.70)	0.507	0.22 (0.19–0.25)	0.148	99.82 (98.99–100.00)	>0.999	3.50 (3.28–3.74)	**<0.001**	15.93 (13.66–18.57)	**<0.001**
	Control	54.43 (50.26–58.56)		0.25 (0.22–0.29)		99.83 (99.03–100.00)		2.65 (2.49–2.82)		10.51 (9.08–12.16)	
14	Experiment	99.64 (98.69–99.96)	0.724	1.89 (1.78–2.01)	0.231	100.00 (99.33–100.00)	-	14.30 (13.43–15.23)	**0.040**	7.57 (6.90–8.31)	0.617
	Control	99.30 (98.23–99.81)		2.00 (1.87–2.14)		100.00 (99.36–100.00)		15.70 (14.74–16.71)		7.85 (7.06–8.73)	
18C	Experiment	42.26 (38.09–46.51)	0.113	0.20 (0.17–0.23)	0.051	99.64 (98.69–99.96)	0.318	1.61 (1.52–1.71)	**<0.001**	8.15 (7.07–9.39)	**0.008**
	Control	46.96 (42.82–51.13)		0.24 (0.21–0.26)		98.96 (97.74–99.62)		2.53 (2.37–2.70)		10.74 (9.30–12.42)	
19A	Experiment	92.90 (90.42–94.90)	0.148	0.81 (0.76–0.87)	**0.006**	100.00 (99.33–100.00)	-	6.48 (6.05–6.93)	**<0.001**	7.97 (7.23–8.78)	**<0.001**
	Control	94.96 (92.84–96.60)		0.92 (0.87–0.98)		100.00 (99.36–100.00)		4.45 (4.21–4.70)		4.81 (4.39–5.27)	
19F	Experiment	87.80 (84.76–90.42)	0.085	0.69 (0.64–0.74)	0.067	100.00 (99.33–100.00)	-	12.73 (11.93–13.58)	**<0.001**	18.52 (16.77–20.46)	**<0.001**
	Control	90.96 (88.31–93.17)		0.75 (0.70–0.80)		100.00 (99.36–100.00)		4.24 (4.02–4.48)		5.64 (5.13–6.20)	
23F	Experiment	45.36 (41.13–49.63)	0.112	0.27 (0.25–0.30)	0.091	99.82 (98.99–100.00)	0.398	3.49 (3.26–3.74)	0.474	12.72 (11.23–14.40)	0.087
	Control	50.09 (45.92–54.25)		0.31 (0.28–0.34)		99.30 (98.23–99.81)		3.37 (3.13–3.62)		10.93 (9.68–12.34)	

Note: Significant differences (*p* < 0.05) are shown in bold.

**Table 4:** Type-specific seropositive rates, GMC and GMI of IgG response pre- and post-booster vaccination (PPS2).

		Pre-Booster Vaccination				Post-Booster Vaccination					
Serotype	Group	Seropositive Rate % (95% CI)	*p*	GMC (95% CI)	*p*	Seropositive Rate % (95% CI)	*p*	GMC (95% CI)	*p*	GMI (95% CI)	*p*
1	Experiment	97.45 (95.77–98.60)	0.171	0.93 (0.89–0.98)	0.510	100.00 (99.33–100.00)	-	3.85 (3.63–4.10)	0.126	19.85 (17.34–22.71)	0.644
	Control	96.00 (94.06–97.45)		0.96 (0.91–1.00)		100.00 (99.36–100.00)		4.11 (3.89–4.35)		19.00 (16.72–21.58)	
3	Experiment	44.36 (40.16–48.63)	**<0.001**	0.34 (0.32–0.36)	**<0.001**	99.27 (98.15–99.80)	**<0.001**	1.33 (1.26–1.41)	**<0.001**	22.72 (19.69–26.22)	**<0.001**
	Control	24.52 (21.06–28.25)		0.24 (0.22–0.25)		96.00 (94.06–97.45)		0.92 (0.87–0.96)		14.78 (12.77–17.10)	
4	Experiment	83.09 (79.69–86.13)	0.121	0.59 (0.56–0.63)	0.107	100.00 (99.33–100.00)	-	3.35 (3.14–3.57)	**<0.001**	65.49 (55.33–77.53)	**<0.001**
	Control	79.48 (75.94–82.71)		0.55 (0.52–0.59)		100.00 (99.36–100.00)		2.57 (2.42–2.74)		40.66 (34.52–47.89)	
5	Experiment	64.36 (60.20–68.37)	**<0.001**	0.37 (0.34–0.40)	**<0.001**	99.27 (98.15–99.80)	>0.999	1.49 (1.40–1.58)	**0.007**	9.82 (8.61–11.21)	0.424
	Control	82.09 (78.70–85.14)		0.52 (0.49–0.55)		99.13 (97.98–99.72)		1.66 (1.57–1.75)		9.14 (8.10–10.30)	
6A	Experiment	90.91 (88.19–93.18)	**0.032**	0.75 (0.71–0.80)	**<0.001**	100.00 (99.33–100.00)	-	4.55 (4.25–4.87)	**<0.001**	21.39 (18.72–24.43)	0.091
	Control	94.26 (92.03–96.02)		0.96 (0.90–1.02)		100.00 (99.36–100.00)		6.07 (5.66–6.50)		25.14 (22.02–28.71)	
6B	Experiment	97.45 (95.77–98.60)	0.245	1.16 (1.09–1.23)	**<0.001**	100.00 (99.33–100.00)	-	7.66 (7.15–8.21)	**<0.001**	18.40 (16.37–20.68)	0.350
	Control	98.43 (97.05–99.28)		1.39 (1.32–1.47)		100.00 (99.36–100.00)		9.44 (8.78–10.14)		19.92 (17.68–22.45)	
7F	Experiment	100.00 (99.33–100.00)	0.145	2.48 (2.34–2.63)	**<0.001**	100.00 (99.33–100.00)	-	10.28 (9.68–10.92)	**<0.001**	49.63 (43.14–57.09)	**<0.001**
	Control	99.30 (98.23–99.81)		1.38 (1.31–1.44)		100.00 (99.36–100.00)		4.94 (4.66–5.23)		21.70 (18.90–24.91)	
9V	Experiment	92.36 (89.82–94.44)	**0.023**	0.87 (0.81–0.93)	**<0.001**	100.00 (99.33–100.00)	-	3.70 (3.46–3.95)	**0.015**	16.81 (14.43–19.58)	**0.021**
	Control	88.35 (85.44–90.85)		0.69 (0.64–0.74)		100.00 (99.36–100.00)		3.31 (3.11–3.52)		13.13 (11.37–15.16)	
14	Experiment	100.00 (99.33–100.00)	-	5.30 (5.03–5.59)	0.560	100.00 (99.33–100.00)	-	17.80 (16.74–18.93)	0.186	9.40 (8.65–10.22)	0.982
	Control	100.00 (99.36–100.00)		5.42 (5.16–5.69)		100.00 (99.36–100.00)		18.83 (17.79–19.94)		9.42 (8.62–10.28)	
18C	Experiment	56.73 (52.47–60.91)	**<0.001**	0.35 (0.32–0.38)	**<0.001**	100.00 (99.33–100.00)	0.500	2.23 (2.10–2.38)	**<0.001**	11.27 (9.72–13.07)	0.141
	Control	78.09 (74.48–81.40)		0.54 (0.50–0.59)		99.65 (98.75–99.96)		3.09 (2.88–3.30)		13.12 (11.42–15.07)	
19A	Experiment	100.00 (99.33–100.00)	-	2.41 (2.27–2.56)	**<0.001**	100.00 (99.33–100.00)	-	14.20 (13.27–15.20)	**<0.001**	17.44 (15.86–19.18)	**<0.001**
	Control	100.00 (99.36–100.00)		2.00 (1.89–2.12)		100.00 (99.36–100.00)		10.42 (9.79–11.09)		11.27 (10.31–12.33)	
19F	Experiment	100.00 (99.33–100.00)	-	3.47 (3.26–3.70)	**<0.001**	100.00 (99.33–100.00)	-	15.74 (14.75–16.80)	**<0.001**	22.90 (20.78–25.23)	**<0.001**
	Control	100.00 (99.36–100.00)		1.71 (1.61–1.81)		100.00 (99.36–100.00)		6.94 (6.55–7.35)		9.23 (8.43–10.09)	
23F	Experiment	94.55 (92.30–96.29)	0.935	1.05 (0.98–1.11)	0.409	100.00 (99.33–100.00)	-	7.61 (7.08–8.18)	0.432	27.66 (24.43–31.32)	0.437
	Control	94.43 (92.23–96.16)		1.08 (1.02–1.15)		100.00 (99.36–100.00)		7.94 (7.35–8.59)		25.79 (22.73–29.25)	

Note: Significant differences (*p* < 0.05) are shown in bold.

**Table 5:** GMT and percentage of participants with OPA ≥ 1:8 pre- and post-primary and booster vaccination (PPS-O2).

		Post-Primary Vaccination				Post-Booster Vaccination			
Serotype	Group	GMT (95% CI)	*p*	Percentage % (95% CI)	*p*	GMT (95% CI)	*p*	Percentage % (95% CI)	*p*
1	Experiment	64.13 (53.85–76.37)	**0.045**	99.26 (95.97–99.98)	>0.999	201.76 (167.99–242.32)	0.552	100.00 (97.32–100.00)	-
	Control	84.15 (68.80–102.92)		100.00 (97.32–100.00)		218.77 (179.76–266.25)		100.00 (97.32–100.00)	
3	Experiment	274.81 (234)	<0.001	99.26 (95.97–99.98)	>0.999	388.85 (334.67–451.80)	**<0.001**	100.00 (97.32–100.00)	-
	Control	176.66 (150.23–207.75)		98.53 (94.79–99.82)		266.52 (229.73–309.20)		100.00 (97.32–100.00)	
4	Experiment	1676.35 (1457.16–1928.50)	0.957	100.00 (97.32–100.00)	-	3745.48 (3223.64–4351.78)	0.205	100.00 (97.32–100.00)	-
	Control	1667.15 (1438.41–1932.27)		100.00 (97.32–100.00)		3245.96 (2752.51–3827.86)		100.00 (97.32–100.00)	
5	Experiment	100.57 (72.89–138.77)	**<0.001**	93.38 (87.81–96.93)	**0.007**	241.31 (173.42–335.78)	**<0.001**	94.12 (88.74–97.43)	**0.012**
	Control	480.13 (406.81–566.67)		100.00 (97.32–100.00)		572.43 (496.14–660.43)		100.00 (97.32–100.00)	
6A	Experiment	3413.41 (2698.95–4316.99)	**0.002**	99.26 (95.97–99.98)	>0.999	8310.48 (6841.70–10094.57)	0.106	100.00 (97.32–100.00)	-
	Control	5487.89 (4549.84–6619.35)		100.00 (97.32–100.00)		10256.74 (8673.02–12129.66)		100.00 (97.32–100.00)	
6B	Experiment	2396.08 (2012.45–2852.84)	0.518	100.00 (97.32–100.00)	>0.999	8249.30 (6686.77–10176.96)	0.656	100.00 (97.32–100.00)	-
	Control	2636.91 (2085.33–3334.38)		99.26 (95.97–99.98)		8832.50 (7095.36–10994.93)		100.00 (97.32–100.00)	
7F	Experiment	20448.46 (17604.30–23752.12)	**0.002**	100.00 (97.32–100.00)	-	21546.00 (18777.59–24722.56)	**<0.001**	100.00 (97.32–100.00)	-
	Control	14679.18 (12669.71–17007.35)		100.00 (97.32–100.00)		11435.93 (10049.84–13013.19)		100.00 (97.32–100.00)	
9V	Experiment	1596.55 (1373.24–1856.17)	0.579	100.00 (97.32–100.00)	-	1929.21 (1663.58–2237.25)	0.215	100.00 (97.32–100.00)	-
	Control	1497.49 (1261.59–1777.50)		100.00 (97.32–100.00)		2221.24 (1876.91–2628.73)		100.00 (97.32–100.00)	
14	Experiment	3730.31 (2998.52–4640.69)	0.665	99.26 (95.97–99.98)	>0.999	3639.87 (3080.09–4301.38)	0.685	100.00 (97.32–100.00)	-
	Control	3482.40 (2778.24–4365.04)		99.26 (95.97–99.98)		3836.43 (3159.09–4659.00)		100.00 (97.32–100.00)	
18C	Experiment	1123.33 (932.96–1352.54)	**<0.001**	100.00 (97.32–100.00)	-	2690.79 (2219.45–3262.24)	0.658	100.00 (97.32–100.00)	-
	Control	2322.84 (1922.91–2805.95)		100.00 (97.32–100.00)		2845.63 (2428.41–3334.55)		100.00 (97.32–100.00)	
19A	Experiment	2721.42 (2306.37–3211.17)	**0.007**	100.00 (97.32–100.00)	-	5563.22 (4839.25–6395.50)	0.168	100.00 (97.32–100.00)	-
	Control	1939.16 (1617.63–2324.60)		100.00 (97.32–100.00)		4829.45 (4170.44–5592.59)		100.00 (97.32–100.00)	
19F	Experiment	1415.96 (1190.73–1683.79)	**<0.001**	99.26 (95.97–99.98)	>0.999	2394.17 (2077.35–2759.30)	**<0.001**	100.00 (97.32–100.00)	-
	Control	536.41 (456.42–1683.79)		99.26 (95.97–99.98)		1171.53 (1025.41–1338.46)		100.00 (97.32–100.00)	
23F	Experiment	7555.55 (6004.71–9506.93)	0.686	99.26 (95.97–99.98)	>0.999	18517.82 (15682.05–21866.40)	0.212	100.00 (97.32–100.00)	-
	Control	7047.07 (6161.18–8641.94)		99.26 (95.97–99.98)		15690.48 (12818.02–19206.64)		100.00 (97.32–100.00)	

Note: Significant differences (*p* < 0.05) are shown in bold.

## Data Availability

The data presented in this study are available upon request from the corresponding authors.
